# Bridging “Office-Based Care” With the “Virtual Practice Care Model”: Evolving Care for Chronic Kidney Disease Patients in the COVID-19 Pandemic—And Beyond

**DOI:** 10.3389/fmed.2020.568201

**Published:** 2020-11-09

**Authors:** Bingbin Zhao, Lei Zhang, Peili Ji, Jianfeng Lin, Jianfang Han, Jiaying Li, Zijuan Zhou, Haiyun Wang, Ling Qiu, Xia Hong, Winfred Williams, Limeng Chen

**Affiliations:** ^1^Department of Nephrology, Peking Union Medical College Hospital, Peking Union Medical College, Chinese Academy of Medical Sciences, Beijing, China; ^2^Department of Nephrology, Qinghai Provincial People's Hospital, Xining, China; ^3^Department of Clinical Laboratory, Peking Union Medical College Hospital, Peking Union Medical College, Chinese Academy of Medical Sciences, Beijing, China; ^4^Department of Psychological Medicine, Peking Union Medical College Hospital, Peking Union Medical College, Chinese Academy of Medical Sciences, Beijing, China; ^5^Department of Nephrology, Massachusetts General Hospital and Harvard Medical School, Boston, MA, United States

**Keywords:** chronic kidney disease, telemedicine, coronavirus, psychological stress, patient care

## Abstract

Since the outbreak of the coronavirus epidemic, the “virtual” telemedicine has become a critical substitute for patient-provider interactions. However, virtual encounters often face challenges in the care of patients in high-risk categories such as chronic kidney disease (CKD) patients. In this study, we explore the patient's satisfaction and the practical effects of a newly established telemedicine program on CKD patients' care during the COVID-19 pandemic. Based on a prior version of an online patient care platform established in 2017, we developed a customized and improved online telemedicine program designed to specifically address the challenges emerging from the pandemic. This included an online, smart phone-based strategy for triage and medical care delivery and psychological support. We invited a total of 278 CKD patients to join the new platform during the pandemic. The subjects in group A were patients utilizing our old online CKD system and were historical users registered at least 3 months before the pandemic. A pilot survey interrogating medical and psychological conditions was conducted. Feedback on the program as well as a psychological assessment were collected after 1 month. In total, 181 patients showed active responses to the program, with 289 person-time medical consultations occurring during the study. The virtual care program provided a rapid triage for 17% (30 out of 181) patients, with timely referral to in-patient medical encounters for their worsening medical conditions or severe psychological problems. Nearly all patients (97.4%) believed the program was helpful. The number of symptoms (OR 1.309, 95%CI 1.113–1.541; *P* = 0.001) and being enrolled during the pandemic (OR 3.939, 95% CI 1.174–13.221; *P* = 0.026) were associated with high stress. During the follow-up, the high-stress CKD group at baseline showed a significant decrease in avoidance score (6.9 ± 4.7 vs. 9.8 ± 1.9, *P* = 0.015). In conclusion, during the pandemic, we established an online telemedicine care program for CKD patients that provides a rapid triage function, effective CKD disease management, and potentially essential psychological support.

## Introduction

Since the start of the coronavirus pandemic, by April 2020 more than three million confirmed coronavirus disease 2019 (COVID-19) cases have been reported worldwide. In the midst of this healthcare crisis, a number of strategies have emerged to develop best practices for the safe delivery of healthcare, not only to safeguard patients but also to protect healthcare workers, including first responders (1). A key feature of new, emerging healthcare delivery paradigms is the radical change in traditional management patterns for acute and chronic disease (2). Thus, the number and frequency of non-urgent patient encounters such as elective surgeries (e.g., knee replacement), routine diagnostic procedures (e.g., colonoscopy), routine health maintenance follow-up visits, and even new medical consultations have seen dramatic reductions. In many centers of care around the world, with the exception of medical emergencies, during the current period of social distancing, with the exception of medical emergencies, face-to-face medical encounters have been almost entirely eliminated.

For chronic kidney disease (CKD), one of the most common public health problems and one often associated with high rates of comorbidity and mortality, these changing care patterns present special challenges. Frequently, patients with CKD have acute exacerbations or even life-threatening conditions such as acute heart failure and severe hyperkalemia (3). At Peking Union Medical College Hospital (PUMCH) in Beijing, China, more than 60% of patients presenting for medical attention come from remote areas of the country. Because of the coronavirus outbreak, in the first quarter of 2020, the year-over-year number of CKD outpatient visits of one group in the PUMCH renal division had decreased by 50%.

In the context of the burden of chronic disease and this pandemic, one area of particular concern for CKD patients is psychosocial well-being. It is well-documented that CKD patients are more prone to anxiety, depression, and other psychiatric disorders than those with other chronic illnesses (4). The psychosocial burden in CKD is manifested by a decrease in health-related quality of life measures (e.g., as measured by the CDC HRQoL survey) and has been linked to increased mortality risk (5, 6). As examples, when measurements of post-traumatic stress disorder (PTSD) and other event-related stressors have been measured, the degree of severity in CKD patients was more severe and lasted longer than in the general population (7, 8). Taking the example of PTSD, this psychiatric disorder is generally considered to be precipitated by a severely traumatic event or series of events and characterized by intrusive images and thoughts, avoidance or numbing behaviors, and feelings of hyperarousal. Stereotypically, anger outbursts and loss of self-control have been classic descriptors. Because of the unforeseen severe social and economic devastation brought on by the COVID-19 pandemic and the deep disruption of normal daily life, as well as the inherent fear of contracting a life-threatening illness, PTSD has become a focus of attention similar to its emergence during the severe acute respiratory syndrome (SARS) pandemic of 2002–2004 (9). We posit that event-related distress in CKD patients is currently severely underestimated during the COVID-19 pandemic. This may become the basis of a global challenge to change healthcare delivery systems in response to the urgent need for psychological support services, particularly, but not limited to CKD patients, as well as for first-line responders and medical workers providing care for those afflicted (10).

In this study, in the context of the current pandemic, during the 3-month period from February to April 2020, we explored the outcomes after provision of free telemedicine services for 278 CKD patients in China. We hypothesized that this newly established telemedicine program would enable nephrologists and psychologists to efficiently provide better stratification and management of patients online, timely detection of critically ill patients, and provide much-needed psychological assistance to evaluate and reduce stress responses in vulnerable CKD cohorts.

## Materials and Methods

### Online CKD Care via Smartphone

**Figure 1:**
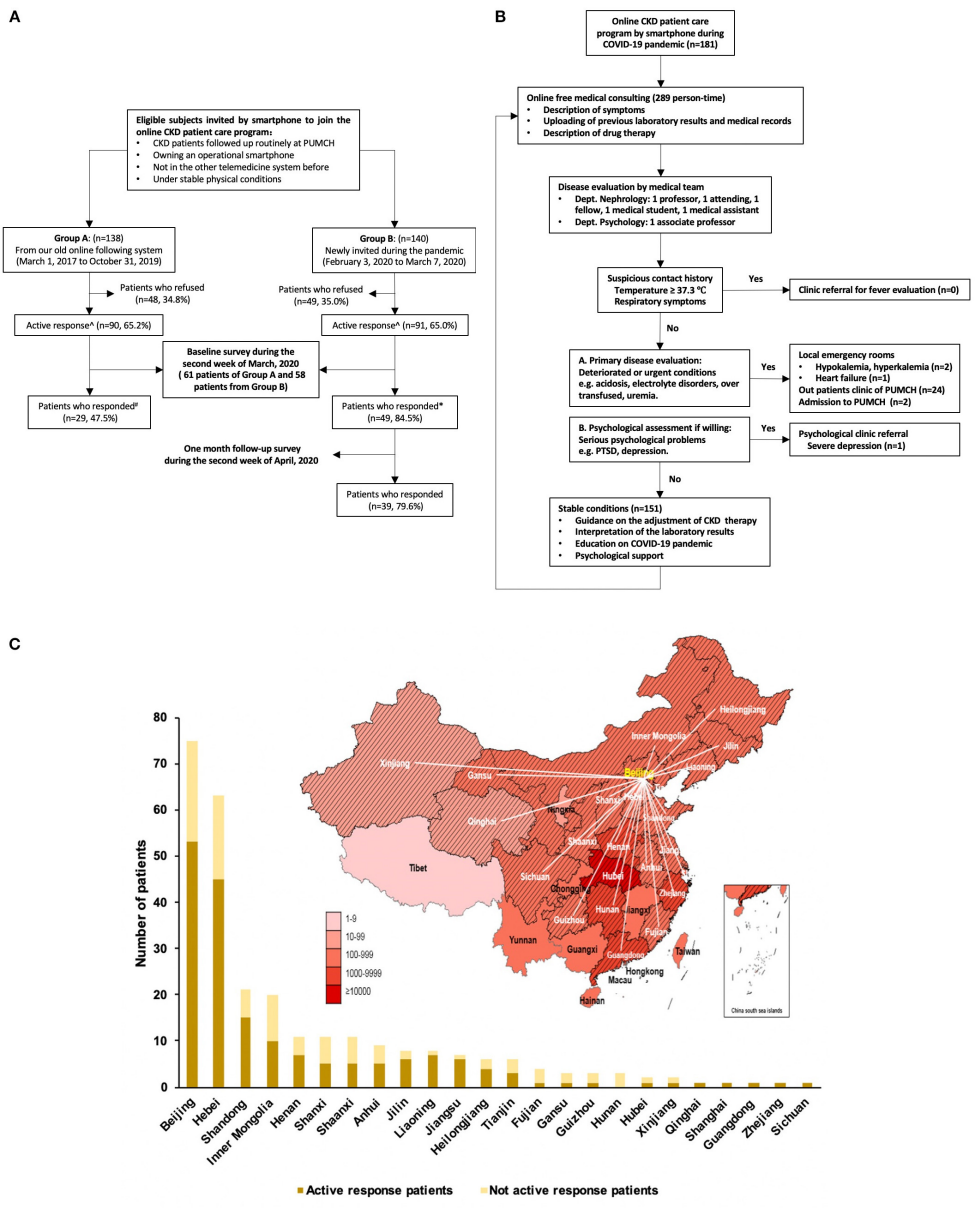
Flowchart of study design **(A)**, the strategy of the online CKD patient care program of CKD patients **(B)**, and the patients' distribution during COVID-19 pandemic **(C)**. **(A)** Active response meant the invited patients agreed to join the online care program. ^#^*There were 61 patients from group A and 58 patients from group B, who received questionnaires. **(C)** The gradation of red color illustrated severity of the pandemic of COVID-19 on April 19, 2020. The shaded areas represent the regions of origin of patient enrollees. CKD, chronic kidney disease; PTSD, post-traumatic stress disorder; PUMCH, Peking Union Medical College Hospital.

Beginning in 2017, providers at PUMCH began using a smartphone application, called “Clipboard of Medicine” (Apricot Forest Incorporated Company, Beijing, China) to provide follow up for routine CKD patients at PUMCH. The platform is composed of a patient side, physician side, and a remote server, connected through cellular networks and smartphone (Supplementary Figure 1). In addition to medical consultation related to clinic visits, short passages related to kidney health were sent to patients every 1–3 months. We invited a total of 278 CKD patients to join our new modified system during the coronavirus pandemic. The subjects of Group A were patients followed on our old online following system from March 1, 2017 to October 31, 2019 (*n* = 138). The newly invited patients during the outbreak of the COVID-19 pandemic (February 3, 2020 to March 7, 2020) were enrolled in Group B (*n* = 140). All patients carried a diagnosis of CKD, and were followed up routinely at the outpatient clinic of PUMCH. Enrollees were physically stable, had a fully functional smartphone and, prior to the study, were not followed by other telemedicine methods. If the patients agreed to join the online care program, then they entered the active response group. All patients had to provide electronic informed consent before joining the platform (Figure 1A). Appointment information, patient demographics and clinical data were collected utilizing the PUMCH digital, electronic medical records system.

The process of online management during the pandemic is shown in Figure 1B. If the patient's symptom complex suggested the possibility of SARS-COV-2 infection, they were referred for evaluation, either to a dedicated clinic or the emergency room (ER). Patients who were in a deteriorated or emergently ill state were immediately triaged to the ER, either at PUMCH or other local hospitals. Stable patients received routine evaluation and management, including interpretation of laboratory test results, treatment recommendations and guidelines, education on the COVID-19 pandemic, and psychological support if needed. For patients with a high IES-R score, we provided public health information on preventative measures to be taken against COVID-19 infection (sponsored by PUMCH at “https://ncov-h5.yibaomd.com/ncov/v2?from=timeline&isappinstalled=0”), continued evaluation of their emotional status via other self-administered scales, and recommended professional mental health referral where indicated.

CKD patients who were followed by our group but not using the smartphone application were selected as the controls. Due to the pandemic, these patient's last visit to PUMCH was at least 3 months prior to study initiation. For analysis, both baseline and follow-up estimated GFR (eGFR) were included for both the online telemedicine intervention and control group. And eGFR within 3 months prior to March 7, 2020 was used as a baseline for comparison between the two study groups.

### Pilot Study Questionnaire

We conducted a pilot survey via an online questionnaire to evaluate the psychological status of patients by the IES-R scale, focusing on subjective distress caused by the COVID-19 pandemic. During the second week of March 2020, an electronic version of the baseline questionnaire was sent to both group A and B patients through an online survey platform (“SurveyStar,” Changsha Ranxing Science and Technology, Shanghai, China).

The aims of the questionnaire were: (1) to evaluate the pandemic related distress; (2) to explore the relationship between chief symptoms, signs, and the level of distress; (3) to figure out other factors correlated with the degree of distress, including self-rating health status, difficulties in drug purchasing, and medical examination. Thus, this structured patient questionnaire consisted of several categories: (1) Demographic information, including age, gender, and home location. (2) Clinical data, including recent serum creatinine (CKD stage based on the CKD-EPI equation) (11), and chief symptoms and signs over 2 weeks. The Supplementary Table 4 shows the 17 common symptoms of CKD patients based on previous studies. They are fatigue, weakness, poor appetite, palpitation, dizziness, fainting, muscle weakness, cramps, restless legs, pain, difficult sleeping, diarrhea, constipation, shortness of breath, nausea, vomiting, and itching; each item rates the frequency of the symptom by a 5-point scale, from 0 (not at all) to 4 (all the time) (12, 13). (3) Self-rated health status (better or worse) throughout the period of evaluation compared with the previous year. (4) Health service access includes drug availability and medical examination during the pandemic, and (5) finally, a measure of psychological distress by the Impact of Event Scale-Revised (IES-R).

Psychological distress was measured by the IES-R survey, a 22-item instrument which in previous studies has proved to have internal consistency, construct, and criteria validity (14, 15). The total score of IES-R consists of 3 subscales: intrusion, hyperarousal, and avoidance (16). Each item rates the frequency of the symptom during the period under study by a 5-point scale, from 0 (not at all) to 4 (extremely). High-stress was defined as an IES-R total score equal to or <20 (17).

After 1-month of online consultation and support, patients in group B, newly recruited patients who responded to the baseline questionnaire, were asked to give feedback on the online care program during the second week of April 2020. The follow-up survey included a rating of the value of the medical services (Figure 1A).

The study was approved by the ethics committee of Peking Union Medical College Hospital (S-K1066). It followed the principles in the Declaration of Helsinki. All patients enrolled in the online care program gave digital informed consent at the time of registration of the smartphone application (Clipboard of Medicine).

### Statistical Analyses

We presented the continuous variables as mean (SD) or median (interquartile range), while the categorical data as number (percentage). A two-sample *t*-test was used to compare the difference in age and IES-R scores of the different subgroups. We also analyzed the numbers of symptoms in high-stress and low-stress groups by the Mann-Whitney test. The chi-square or Fisher's exact test was used to analyze the proportions of sex, area, disease category, CKD stage, fatigue, weakness, palpitation, poor appetite, poor self-rated health status, difficulty in purchasing drugs, and difficulty obtaining routine lab tests. We used Spearman correlation to evaluate the relationship between the symptoms and stress levels, and performed binary logistic regression models to examine the independent risk factors of high stress. The full model included six factors: age, sex (male), number of symptoms, enrolled during pandemic, difficulty in obtaining medicines, and difficulty in obtaining medical evaluation, followed by the forward selection (Likelihood Ratio) to build the final model. A paired *t*-test was processed to compare the decline of kidney function in patients with or without telemedicine, IES-R score (the total score, intrusion score, hyperarousal score, and avoidance score) before and 1 month after joining the online program. The absolute reduction values of IES-R score (the total score, intrusion score, hyperarousal score, and avoidance score) after a 1-month follow-up were compared by two sampled *t*-test between the high-stress group and low-stress group. The data were analyzed using appropriate procedures in SPSS 17.0 statistical software (SPSS, Chicago, IL). *P*-values were two-sided, with *P* < 0.05 considered statistically significant.

## Results

### Online Triage and Consulting During COVID-19 Pandemic

The 278 invited patients came from 24 provinces of mainland China, with 73.0% coming from areas outside Beijing (Figure 1C). A total of 181 patients were finally enrolled in the online program; half of them enrolled during the time of the COVID-19 pandemic and half were followed at least 3 months before the pandemic hit. Another 97 patients refused our invitation. There was no difference in the age, gender, home location, primary disease, and CKD stages between groups with or without active response (Table 1), with the exception of the lesser number of enrollees with a diagnosis of diabetic kidney disease (DKD) in the active response group (8.7 vs. 18.6%; *P* = 0.017).

**Table 1:** General information of active response patients to the online CKD patient care program.

	**Active response patients (*n* = 181)**	**No active response patients (*n* = 97)**	***P-*value**
Age (years)	41.1 ± 14.7^*^	41.2 ± 17.0	0.951
Male (%)	78 (44.6%)^#^	53 (54.6%)	0.111
**Area (%)**			
Outside Beijing	128 (70.7%)	75 (77.3%)	0.237
Rural areas	72 (41.6%)^**^	50 (51.5%)	0.116
**Disease category (%)**			
CGN	57 (32.9%)^**^	28 (28.9%)	0.488
CIN	92 (53.2%)^**^	51 (52.6%)	0.924
DKD	15 (8.7%)^**^	18 (18.6%)	0.017
Hypertension	61 (35.3%)^**^	32 (33.0%)	0.706
Other	106 (61.3%)^**^	62 (63.9%)	0.667
**CKD stage (%)**			
1	96 (55.5%)^**^	54 (55.7%)	0.877
2	24 (13.9%)^**^	15 (15.5%)	
3	29 (16.8%)^**^	12 (12.4%)	
4	17 (9.8%)^**^	11 (11.3%)	
5	7 (4.0%)^**^	5 (5.2%)	

*Outside Beijing, living at more than 80 kilometers outside metropolitan Beijing*.

* *n = 172*,

# *n = 175*,

** *n = 173. CGN, chronic glomerulonephritis; CIN, chronic interstitial nephropathy; CKD, chronic kidney disease; DKD, diabetic kidney disease*.

During six weeks, our team provided a total of 289 person-times of medical consults (one consult from one patient was defined as one person-time medical consult). Most of them (98.9%) entailed routine medical care services, such as explanation of a medical condition and lab results, fulfilling prescriptions, dietary guidance, and other health maintenance issues.

Although no patient in this study was suspected of having SARS-COV-2 infection, the final triage decisions were based on symptoms, physical signs, and recent laboratory results. There were 151 (83.4%) stable patients. Three patients (1.7%) were referred to local emergency rooms because of hypokalemia, hyperkalemia, and heart failure. Twenty-four patients (13.3%) were referred to PUMCH CKD outpatient clinics and two patients (1.1%) were admitted to PUMCH wards. Finally, one patient (0.6%) was referred for psychological evaluation and management of severe depression (Figure 1B).

### Evaluation From the Patients

**Figure 2:**
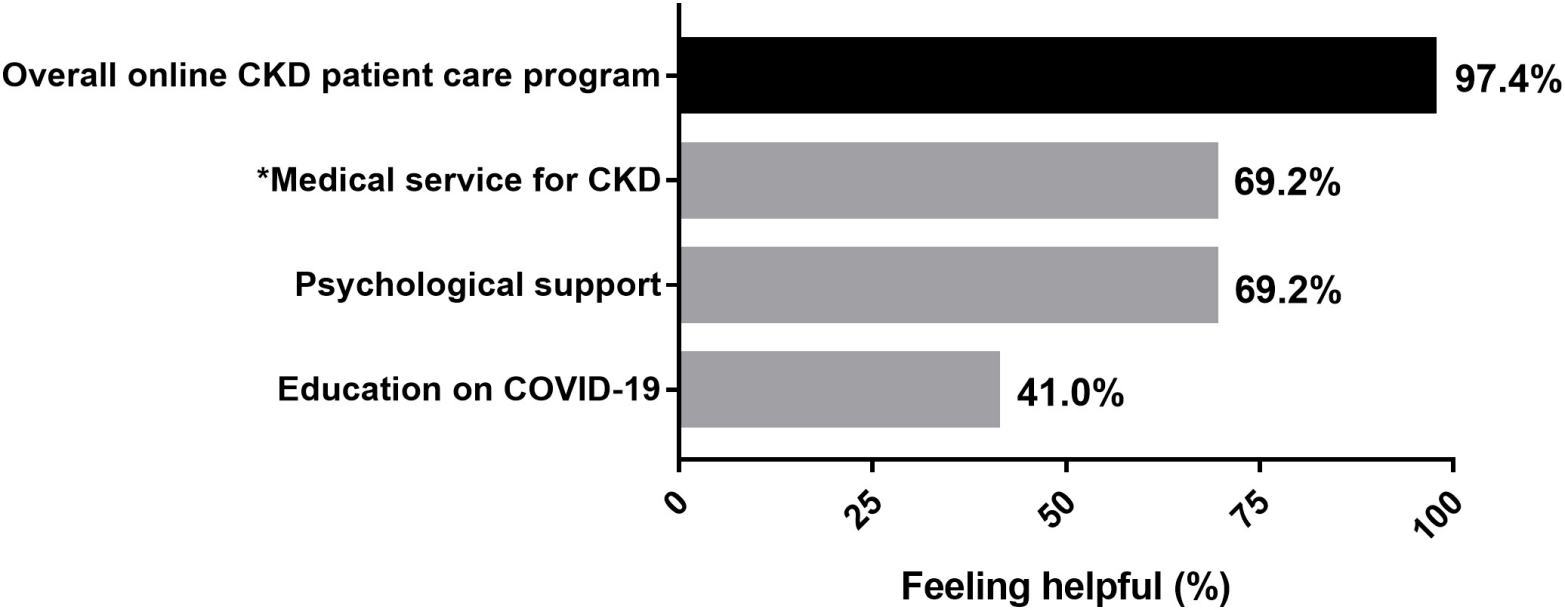
Patient satisfaction with telemedicine services (*n* = 39). A total of 38 (97.4%) respondents believed that this new system of care delivery was effective in at least one aspect, including medical service for kidney disease (69.2%), psychological support (69.2%), and COVID-19 prevention education (41.0%). *The medical service for CKD including evaluation of symptoms, interpretation of laboratory results, adjustment of therapy, guidance on local clinic visits, education on kidney health. CKD, chronic kidney disease.

After 1-month of management, 39 (79.6%) patients in group B responded to our survey for rating of the value of the medical services. There was a high degree of patient satisfaction with online telemedicine services: 97.4% of the patients believed that this new system of care delivery was effective in at least one aspect, including medical service for kidney disease (69.2%), psychological support (69.2%), and COVID-19 prevention education (41.0%, Figure 2). Approximately half of the cohort (48.7%) appreciated both medical services and psychological support; other patients preferred medical (41.0%), or psychological (41.0%) support, and only three patients (7.7%) indicated that COVID-19 prevention education was the most crucial benefit of the program.

### Decline of Kidney Function in Patients With Telemedicine or Without

There was no significant difference in the clinical characteristics, primary disease, and eGFR in both telemedicine and control groups (Supplementary Table 3). During follow-up, eGFR of patients in both groups declined, but only the eGFR of the control group showed a statistically significant decline by paired *t*-test (*P* = 0.036).

### Characteristics of CKD Patients Involved in the Pilot Survey

In total, 78 CKD patients completed the online questionnaire in the second week of March, 2020. Compared with patients who joined the telemedicine platform before the pandemic, the newly enrolled patients were more likely to complete the survey (84.5 vs. 47.5%; *P* < 0.001) and were older (45.2 ± 12.7 vs. 36.3 ± 12.1 years; *P* = 0.003). There was no difference between the IES-R total and subscale scores. The percentage of highly stressed participants joining the telemedicine program in the later cohort (Group B) was 49.0%, higher than patients who joined before the pandemic (Group A) (37.9%; *P* = 0.343) (Supplementary Table 1).

### Risk Factors of High-Stress Patients

Compared with the low-stress patients, the high-stress patients had more symptoms [9.0 (6.0, 11.0) vs. 6.0 (3.0, 9.0); *P* = 0.004], including at least three general symptoms (fatigue, weakness, and palpitations) (Table 2), which correlated well with the IES-R score. Weakness and palpitation were significantly related with all the subscale scores (intrusion, hyperarousal, and avoidance), but poor appetite (*P* < 0.05) and fatigue (*P* < 0.001) only associated with hyperarousal (Supplementary Table 2).

**Table 2:** Characteristics of the patient population—symptoms, health status, and accessibility to medical services between patients with high-stress and low-stress.

	**High-stress (*n* = 35)**	**Low-stress (*n* = 43)**	***P*-value**
Age(years)	43.4 ± 11.1	40.7 ± 14.6	0.370
Male	12 (34.3%)	21 (48.8%)	0.196
**CKD stage**			
1	14 (40.0%)	25 (58.1%)	0.486
2	7 (20.0%)	4 (9.3%)	
3	7 (20.0%)	7 (16.3%)	
4	5 (14.3%)	4 (9.3%)	
5	2 (5.7%)	3 (7.0%)	
Number of symptoms	9.0 (6.0, 11.0)	6.0 (3.0, 9.0)	0.004
**General symptom**			
Fatigue	27 (77.1%)	24 (55.8%)	0.049
Weakness	30 (85.7%)	25 (58.1%)	0.008
Palpitation	20 (57.1%)	15 (34.9%)	0.049
Poor appetite	13 (37.1%)	14 (32.6%)	0.672
Poor self-rated health status	16 (45.7%)	17 (39.5%)	0.583
Difficulty purchasing drugs	20 (57.1%)	20 (46.5%)	0.350
Difficulty obtaining routine lab tests	20 (57.1%)	29 (67.4%)	0.349

The multivariate binary logistic regression model showed that the number of symptoms (odds ratio 1.309, 95% confidence interval 1.113–1.541; *P* = 0.001) and being enrolled during pandemic (odds ratio 3.939, 95% confidence interval 1.174–13.221; *P* = 0.026) independently predict the IES-R, when adjusted by age, gender and difficulty for medical services (Table 3).

**Table 3:** Multivariate logistic analysis of the predictors of high stress by the IES-R scale in CKD patients.

	**Full model**			Final model*		
	**OR**	**95% CI**	***P*-value**	**OR**	**95% CI**	***P*-value**
Age	0.996	0.954–1.039	0.853			
Male	0.738	0.261–2.088	0.567			
Number of symptoms	1.334	1.105–1.612	0.003	1.309	1.113–1.541	0.001
Enrolled during pandemic	5.004	1.113–22.507	0.036	3.939	1.174–13.221	0.026
Difficulty 1	0.685	0.221–2.120	0.512			
Difficulty 2	0.881	0.305–2.546	0.815			

* *Final model: Forward selection (Likelihood Ratio). n = 78. IES-R, impact of event scale-revised; Difficulty 1, difficulty obtaining medicines; Difficulty 2, difficulty obtaining medical evaluation*.

### Follow-Up of Patients

**Figure 3:**
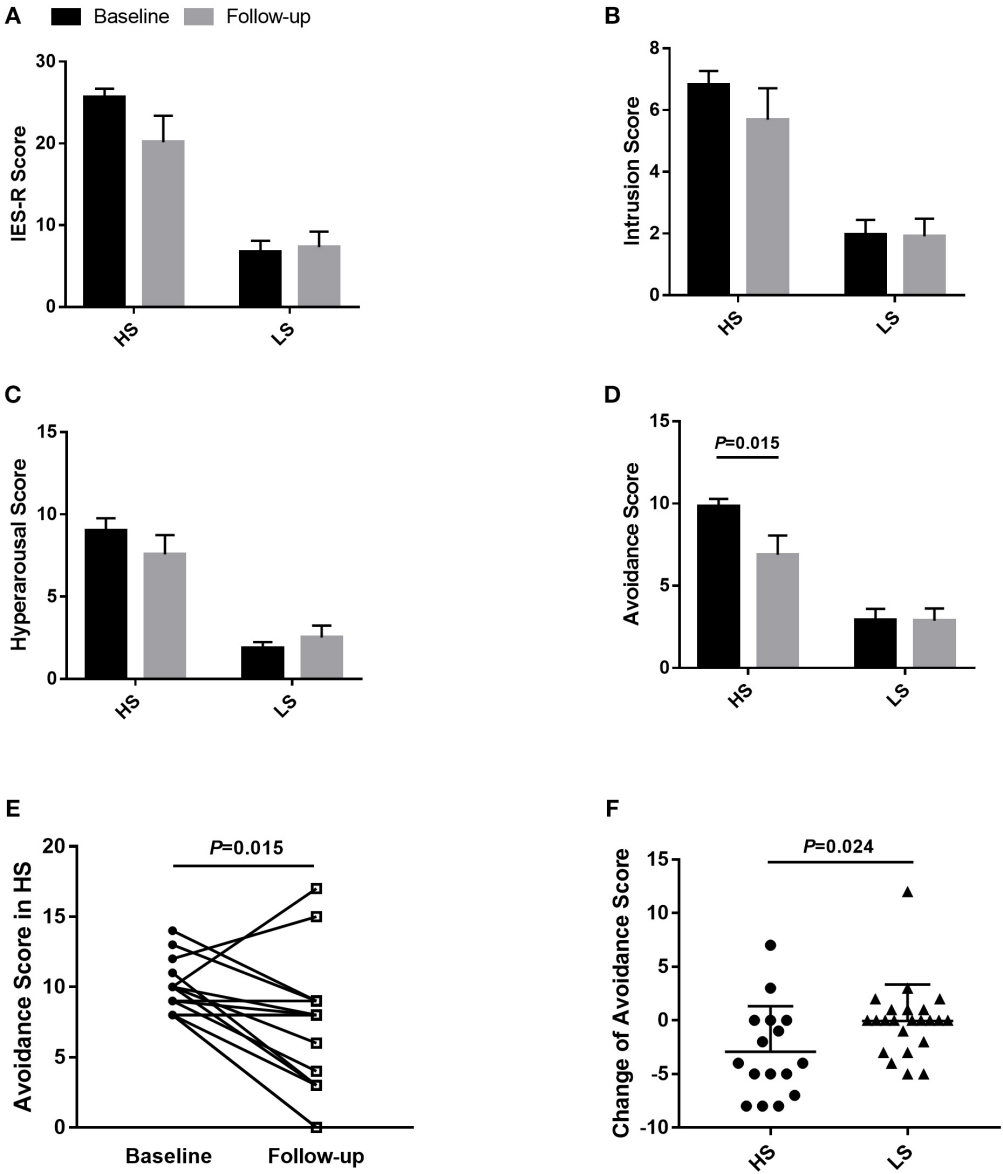
Impact of Event Scale-Revised (IES-R) evaluation of CKD patients at baseline and 1-month follow-up. Comparison of IES-R score **(A)**, intrusion scores **(B)**, hyperarousal score **(C)**, and avoidance score **(D)**, before and 1 month after joining the online program of HS and LS patients. Comparison of avoidance score **(E)** between baseline and follow-up in the HS group, absolute reduction values of avoidance score **(F)** between HS and LS patients. The values in **(A–D)** were shown as mean ± SEM. HS, high-stress group; LS, low-stress group; IES-R, Impact of Event Scale-Revised; SEM, standard error of the mean.

After 1 month, 79.6% of online consulting patients gave feedback. In the high-stress patients, the avoidance score declined significantly (9.8 ± 1.9 vs. 6.9 ± 4.7, *P* = 0.015), while there was no significant change of the total IES-R score, intrusion score, and hyperarousal score both in the high-stress and low-stress patients. The absolute reduction value of avoidance score in the high-stress group was higher than that of the low-stress group (2.9 ± 4.3 vs. 0 ± 3.4, *P* = 0.024) (Figure 3).

## Discussion

The COVID-19 viral pandemic has dramatically altered the landscape of traditional in-person medical care. In order to mitigate the risk of ongoing viral spread, many centers have had to rapidly convert their routine and emergency care to prescreening utilization of telehealth services. This has carried over into nearly all aspects of routine continuity care for outpatient medical practices, primary and subspecialty care, including nephrology (18). For CKD, screening and identification of CKD patients with emergency conditions who may require hospitalized medical service is a critical application and challenge for “virtual” telemedicine encounters. In this study, we demonstrate that the “Online CKD Patient Care Program” (OCCP) based on a smartphone platform can play a critical role in providing essential medical services for CKD patients in the midst of the COVID-19 epidemic. We show evidence that we were able to successfully deploy OCCP for primary disease evaluation, medical triage across a range of medical conditions, effect timely and efficient medical management and, importantly, assess the need for psychological support for the stress responses associated with COVID-19.

First, applying OCCP as a triage function has laid the foundation for patient and provider safety in our CKD clinical practice. Besides the stable patients, we found that 17% of patients were provided a timely referral for in-patient medical encounters including referral to outpatient clinics, the ER, and in-hospital services. Additionally, for patients with CKD, OCCP also showed a potential superiority in disease monitoring and treatment adjustment, which might be beneficial in slowing the progressive decline in kidney function during the pandemic of COVID-19. The successful application of this virtual technology was enormously reassuring in the management of the expected, urgent complications often seen in CKD patients. We would emphasize that its success rests on execution by an experienced multidisciplinary team operating with a unified set of standard operating procedures.

Telemedicine is an innovation for physicians to provide primary care for patients with chronic diseases, with many modalities including telephone calls, electronic messaging, and video communications through platforms in smartphones or websites (19). This new access to patients is efficient, convenient, and cost-saving (20), and promotes patient safety in high-risk patients, such as advanced CKD patients with diabetes and hypertension. Recent reports have begun to make recommendations regarding the application of telehealth practices and framework development in the COVID-19 pandemic (18, 21). However, the online management experience, thus far, especially for CKD applications, appears to be far from sufficient. We have established a fully functional online virtual healthcare platform that, when combined with the PUMCH customized in-hospital electronic medical record (PUMCH PLUS for staff and PUMCH APP for patients), enables not only critical triage functionality, but also an array of services: electronic payment capture for testing by high-resolution imaging, routine laboratory-based testing and assays, and even scheduling of outpatient appointments and booking in-patient beds for hospital admission. Thus, the implementation of OCCP has served as a critical bridge for CKD care between traditional institution-based care and online consultancy.

Secondly, we believe this patient-focused, innovative online care model has also contributed enormously to local and regional preventative health measures. It has functioned to help limit SARS-COV-2 infection by personal preventative health education, providing early detection of suspected infection by symptom assessment, and protecting CKD patients from exposure to potential sources of infection in the hospital cluster by avoiding face-to-face contact in the medical institution. This is of particular importance in view of the observation that worse outcomes have been observed in COVID-19 patients with comorbidities such as CKD, as well as obesity, hypertension, cardiovascular and lung diseases (22, 23).

The third advantage of this telemedicine approach is its potential to aid in reducing the stress response to the pandemic. In this regard, it's noteworthy that as of the first week in May 2020, Blue Cross and Blue Shield of Massachusetts, the largest health maintenance organization (HMO) in that U.S. state, recorded more than 500,000 telehealth visits with patients. Before COVID-19, the average number of telehealth visits recorded by this HMO over an average 6-week period was 5,000. It is of particular significance that the majority of the telehealth visits by their subscribers over the 6-week period just preceding May, were for behavioral health, which is a key focus of this report.

We demonstrate that, at baseline, the percentage of high stress in subjects recruited post-pandemic (Group B) was higher than those who enrolled before the pandemic (Group A) (49.0 vs. 37.9%; *P* = 0.343), although the differences between groups was not statistically significant. However, when adjusted by age, gender, and difficulty with access to medical services, newly enrolled subjects independently predict the IES-R (odds ratio 3.939, 95% confidence interval 1.174–13.221; *P* = 0.026). This indicates that even relatively short-term (3 month) online support can be an essential tool in psychological stress reduction in the face of an emergent public health crisis. To wit: after 1 month, 43.8% of the newly enrolled CKD subjects with high stress recovered to the low-stress group. The most significant changes were observed in the reduction of avoidance symptoms, which have been identified to play a key role in PTSD pathophysiology, and might be the most detrimental symptom cluster compared with intrusion and hyperarousal symptoms (24). The feedback that the CKD cohort gave on the OCCP strongly endorses the importance of the psychological support component: approximately half of the cohort (48.7%) appreciated both medical services and psychological support. The number of physical symptoms was another independent risk factor of psychological distress, and the more severe psychological stress was associated with the higher frequencies of general symptoms, including fatigue, weakness, and palpitation. This finding was consistent with previous studies (7, 25). Thus, addressing psychological distress might aid in alleviating somatic symptoms, which in turn could improve the mental health of CKD patients. Unfortunately, in the follow-up survey, 30.8% of the patients still had a high IES-R score, which suggested high distress levels might persist for substantial periods. This result was consistent with previous research conducted by Hyre et al., which found that 23.8% of hemodialysis patients had PTSD symptoms ~1 year after Hurricane Katrina (8). Other surveys have reported that between 25% and 33% of the general populations met levels of PTSD several months after the outbreak of infectious diseases (26, 27). Follow up monitoring of patient's stress levels may provide critical insights into psychological interventions that may be very crucial for recovery of psychological well-being. Since the components of IES-R score (total score, intrusion score, hyperarousal score, and avoidance score) are correlated within groups, the Bonferroni correction and Benjamini-Hochberg procedure may not be used to conduct the multiple comparisons for the non-independent data. More research is still needed to further explore the psychological status and support services in CKD patients.

In the end, there were some barriers to telemedicine service implementation. First, patients from rural areas were less active responders (Table 1). This may be a function of the varying levels of internet connectivity, such as network coverage, smartphone usage, and educational level; all of these variables would cause difficulties in telemedicine utilization and popularization (18). Secondly, the questionnaire response rate in newly joined patients was higher than those joined before the pandemic (84.5 vs. 47.5%; *P* < 0.001). This suggests that we may need more effective strategies to educate patients on the real potential benefits of online, interactive telemedicine tools. Face-to-face communication was still the best and most effective way to establish a stable and long-term patient-doctor relationship between the medical faculty, patients, and their family members.

Our study has some limitations. As a pilot study, the limited sample size, rush time, low response rate, and the short time of follow-up, might produce bias when compared with the real world CKD population. With time, we feel confident that we will be able to further and more accurately assess the effect of the program on patients' psychological status in the long term. Besides the IES-R scale, other survey instruments that focus on depression and anxiety may reveal further, more comprehensive insights on psychological stressors, the psychological profiles of CKD patients, and aid in refining treatment modalities. Despite these limitations, our study is still the first to explore strategies to bridge the gap between the telemedicine and in-hospital medical care for CKD patients during COVID-19, which may provide important insights to health providers around the world.

## Conclusion

We established an online CKD patient care program that provides rapid triage, effective online-based evaluation and management capability, with a potential beneficial effect on patients' well-being. OCCP as a triage function has laid the foundation for patient and provider safety in our CKD clinical practices and has functioned to provide a critical bridge to patient care continuity during this global public health crisis.

## Data Availability Statement

The original contributions presented in the study are included in the article/Supplementary Materials, further inquiries can be directed to the corresponding author/s.

## Ethics Statement

The studies involving human participants were reviewed and approved by the ethics committee of Peking Union Medical College Hospital (S-K1066). The patients/participants provided their written informed consent to participate in this study.

## Author Contributions

XH, WW, and LC: conceptualization, supervision, and writing—review and editing. LC and LQ: data curation. LC: funding acquisition, project administration, and resources. BZ, LZ, and PJ: formal analysis. BZ, LZ, PJ, JianL, JH, JiayL, ZZ, and HW: investigation. BZ: methodology and writing—original draft. BZ and LZ: software. BZ, LZ, XH, WW, and LC: validation. BZ and JianL: visualization. All authors contributed to the article and approved the submitted version.

## Conflict of Interest

The authors declare that the research was conducted in the absence of any commercial or financial relationships that could be construed as a potential conflict of interest.
