# Supplementary material for: Bridging “Office-Based Care” With the “Virtual Practice Care Model”: Evolving Care for Chronic Kidney Disease Patients in the COVID-19 Pandemic—And Beyond
Source: Front Med (Lausanne). 2020 Nov 9;7:568201. doi: 10.3389/fmed.2020.568201 (PMC7680969; doi:10.3389/fmed.2020.568201)
Supplement: Supplementary file 1 [file Data_Sheet_1.docx]

Supplementary Material

# Supplementary Table 1. General information and IES-R score in the baseline survey.

|  | **Patients joined before COVID-19***  **(n=29)** | **Patients joined after COVID-19****  **(n=49)** | ***P* value** |
| --- | --- | --- | --- |
| Age(years) | 36.3±12.1 | 45.2±12.7 | 0.003 |
| Male(%) | 12(41.4%) | 21(42.9%) | 0.898 |
| Non-Hubei area(%) | 28(96.6%) | 49(100%) | 0.789 |
| IES-R score |  |  |  |
| Total | 16.2±14.7 | 16.5±11.8 | 0.930 |
| Intrusion | 4.4±4.2 | 4.4±3.3 | 0.973 |
| Hyperarousal | 5.7±5.5 | 5.7±4.7 | 0.952 |
| Avoidance | 6.1±5.5 | 6.5±4.6 | 0.791 |
| High stress(%) | 11(37.9%) | 24(49.0%) | 0.343 |

*Patients joined the online management system before COVID-19 pandemic, **Patients joined the online management system after COVID-19 pandemic. Abbreviations: IES-R, Impact of event scale-revised; Non-Hubei, living out of Hubei province of China during COVID-19 pandemic.

**Supplementary Table 2. Correlation between frequencies of general symptoms and IES-R score.**

|  | **IES-R score** | | | |
| --- | --- | --- | --- | --- |
|  | **Total** | **Intrusion** | **Hyperarousal** | **avoidance** |
| Fatigue | 0.240* | 0.179 | 0.367** | 0.130 |
| Weakness | 0.374** | 0.327** | 0.451** | 0.262* |
| Palpitation | 0.330** | 0.324** | 0.345** | 0.303** |
| Poor appetite | 0.206 | 0.150 | 0.246* | 0.178 |

**P* <0.05, ***P* <0.01. Abbreviations: IES-R, Impact of event scale-revised.

**Supplementary Table 3. Comparison of clinical characteristics and eGFR between patients with and without telemedicine.**

|  | **Patients with telemedicine**  **(n=40)** | **Patients without telemedicine**  **(n=95)** | ***P* value** |
| --- | --- | --- | --- |
| Age (years) | 45.0±14.0 | 48.2±15.1 | 0.258 |
| Male (%) | 13(32.5%) | 58(50.5%) | 0.055 |
| Primary disease category (%) |  |  |  |
| CGN | 22(55.0%) | 53(55.8%) | 0.933 |
| DN | 2(5.0%) | 3(3.2%) | 0.985 |
| CIN | 9(22.5%) | 10(10.5%) | 0.068 |
| Other | 23(57.5%) | 61(64.2%) | 0.463 |
| Baseline CKD stage (%) |  |  |  |
| 1 | 13(32.5%) | 20(21.0%) | 0.230 |
| 2 | 4(10.0%) | 25(26.3%) |  |
| 3 | 13(32.5%) | 24(25.3%) |  |
| 4 | 6(15.0%) | 15(15.8%) |  |
| 5 | 4(10.0%) | 11(11.6%) |  |
| Baseline eGFR (ml/min/1.73m2) | 61.6±39.6**^#^** | 56.9±33.3* | 0.513 |
| Follow-up eGFR (ml/min/1.73m2) | 59.5±39.3**^#^** | 54.7±36.6* | 0.500 |

**^#^** Decline of eGFR in the telemedicine group was not statistically significant by paired t-test (*P*=0.065). *eGFR of the control group significantly decreased (*P*=0.036). Abbreviations: CGN, chronic glomerulonephritis; DN, diabetic nephropathy; CIN, chronic interstitial nephropathy; CKD, chronic kidney disease; eGFR, estimated glomerular filtration rate.

**Supplementary Table 4. The structured questionnaire (the translated English version for CKD patients).**

| **Part** | **Details** |
| --- | --- |
| **Part I**  **Demographic data** | 1.Your gender:  A. Male  B. Female  2.Your age: ______  3.Where are you living now: ______ |
| **Part II**  **Clinical data** | 4. What is the result of your last test of serum creatinine: ______  5. Self-rating health status in past 2 weeks compared with that in the last year:  A. Better  B. Worse  6. Symptoms you felt in the past 2 weeks (please choose the right frequency)   \|  \| Not at all  (0) \| Seldom  (1) \| Often  (2) \| Usually  (3) \| All the time  (4) \| \| --- \| --- \| --- \| --- \| --- \| --- \| \| Fatigue \|  \|  \|  \|  \|  \| \| Weakness \|  \|  \|  \|  \|  \| \| Poor appetite \|  \|  \|  \|  \|  \| \| Palpitation \|  \|  \|  \|  \|  \| \| Dizziness \|  \|  \|  \|  \|  \| \| Fainting \|  \|  \|  \|  \|  \| \| Muscle weakness \|  \|  \|  \|  \|  \| \| Cramps \|  \|  \|  \|  \|  \| \| Restless legs \|  \|  \|  \|  \|  \| \| Pain \|  \|  \|  \|  \|  \| \| Difficult sleeping \|  \|  \|  \|  \|  \| \| Diarrhea \|  \|  \|  \|  \|  \| \| Constipation \|  \|  \|  \|  \|  \| \| Short of breath \|  \|  \|  \|  \|  \| \| Nausea \|  \|  \|  \|  \|  \| \| Vomiting \|  \|  \|  \|  \|  \| \| Itching \|  \|  \|  \|  \|  \|   7. Do you have any difficulties in drug purchasing during the pandemic?  A. Yes  B. No  8. Do you have any difficulties in medical examination during the pandemic?  A. Yes  B. No |
| **Part III**  **the Impact of IES-R scale** | 9. How much were you distressed or bothered by each difficulty below during the past 2 weeks with respect to COVID-19   \|  \| Not at all (0) \| A little bit  (1) \| Moderately  (2) \| Quite a bit  (3) \| Extremely  (4) \| \| --- \| --- \| --- \| --- \| --- \| --- \| \| 1. Any reminders brought back feelings about it \|  \|  \|  \|  \|  \| \| 2. I had trouble staying asleep \|  \|  \|  \|  \|  \| \| 3. Other things kept making me think about it \|  \|  \|  \|  \|  \| \| 4. I felt irritable and angry \|  \|  \|  \|  \|  \| \| 5. I avoided letting myself get upset \|  \|  \|  \|  \|  \| \| 6. I thought about it when I didn’t mean to \|  \|  \|  \|  \|  \| \| 7. I felt as if it hadn’t happened or wasn’t real \|  \|  \|  \|  \|  \| \| 8. I stayed away from reminders about it \|  \|  \|  \|  \|  \| \| 9. Pictures about it popped into my mind \|  \|  \|  \|  \|  \| \| 10. I was jumpy and easily startled \|  \|  \|  \|  \|  \| \| 11. I tried not to think about it \|  \|  \|  \|  \|  \| \| 12. I was aware that I still had a lot of feelings \|  \|  \|  \|  \|  \| \| 13. My feelings about it were kind of numb \|  \|  \|  \|  \|  \| \| 14. I found myself acting or feeling like I was back \|  \|  \|  \|  \|  \| \| 15. I had trouble falling asleep \|  \|  \|  \|  \|  \| \| 16. I had waves of strong feelings about it \|  \|  \|  \|  \|  \| \| 17. I tried to remove it from my memory \|  \|  \|  \|  \|  \| \| 18. I had trouble concentrating \|  \|  \|  \|  \|  \| \| 19. Reminders caused me to have physical reactions \|  \|  \|  \|  \|  \| \| 20. I had dreams about it \|  \|  \|  \|  \|  \| \| 21. I felt watchful and on guard \|  \|  \|  \|  \|  \| \| 22. I tried not to talk about it \|  \|  \|  \|  \|  \| |
| **Part IV**  **Feedback on the online care program (for follow-up only)** | 10. What’s your attitude towards the following items?   1. 1）Medical service for CKD (including evaluation of symptoms, interpretation of laboratory results, adjustment of therapy, guidance on local clinic visits, education on kidney health)   A. helpful  B. helpless   1. 2）Psychological support   A. helpful  B. helpless   1. 3）Education on COVID-19   A. helpful  B. helpless |


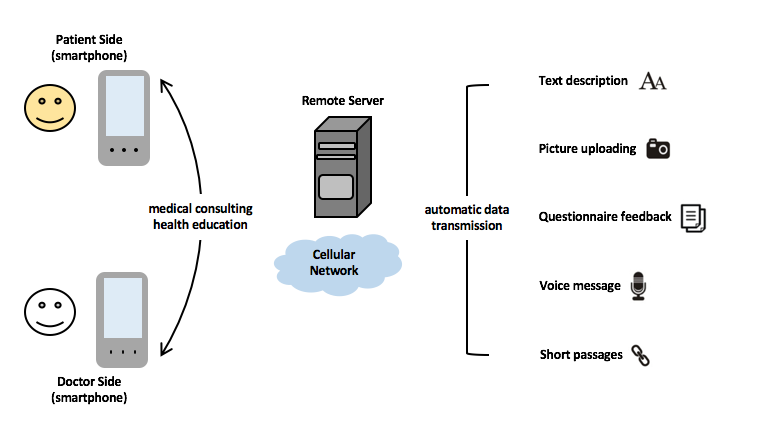


**Supplementary Figure 1. Components of online care platform.**
